# Spatial Baseline Optimization for Spaceborne Multistatic SAR Tomography Systems

**DOI:** 10.3390/s19092106

**Published:** 2019-05-07

**Authors:** Jiuchao Zhao, Anxi Yu, Yongsheng Zhang, Xiaoxiang Zhu, Zhen Dong

**Affiliations:** College of Electronic Science and Engineering, National University of Defense Technology, Changsha 410073, China; zhaojiuchao17@nudt.edu.cn (J.Z.); yu_anxi@nudt.edu.cn (A.Y.); xiaoxiang.z@yahoo.com (X.Z.); dongzhen@nudt.edu.cn (Z.D.)

**Keywords:** spaceborne multistatic SAR tomography (SMS-TomoSAR), spatial baseline optimization, symmetric- geometric model, uniform perturbation

## Abstract

Spaceborne multistatic synthetic aperture radar (SAR) tomography (SMS-TomoSAR) systems take full advantage of the flexible configuration of multistatic SAR in the space, time, phase, and frequency dimensions, and simultaneously achieve high-precision height resolution and low-deformation measurement of three-dimensional ground scenes. SMS-TomoSAR currently poses a series of key issues to solve, such as baseline optimization, spatial transmission error estimation and compensation, and the choice of imaging algorithm, which directly affects the performance of height-dimensional imaging and surface deformation measurement. This paper explores the impact of baseline distribution on height-dimensional imaging performance for the baseline optimization issue, and proposes a feasible baseline optimization method. Firstly, the multi-base multi-pass baselines of an SMS-TomoSAR system are considered equivalent to a group of multi-pass baselines from monostatic SAR. Secondly, we establish the equivalent baselines as a symmetric-geometric model to characterize the non-uniform characteristic of baseline distribution. Through experimental simulation and model analysis, an approximately uniform baseline distribution is shown to have better SMS-TomoSAR imaging performance in the height direction. Further, a baseline design method under uniform-perturbation sampling with Gaussian distribution error is proposed. Finally, the imaging performance of different levels of perturbation is compared, and the maximum baseline perturbation allowed by the system is given.

## 1. Introduction

Knaell proposed two azimuth-range two-dimensional synthetic aperture acquisition techniques—curve SAR (CurviLinear SAR, CLSAR) [1] and SAR tomography (TomoSAR) [2]—in 1994 and 1995, respectively. Compared with interferometric SAR (InSAR) [3], CLSAR and TomoSAR truly qualify as 3D imaging. In TomoSAR, the use of a stack of complex-valued images, by synthesizing apertures along the height direction, makes it possible to separate different scatterers in one range-azimuth resolution cell and achieves height resolution, so as to provide the full 3D scene reflectivity profile in azimuth, range, height, and average velocity deformation [4].

Since the first practical demonstration of TomoSAR [5], it has been successfully applied in many application contexts, such as forestry [6,7], 3D urban reconstruction [8,9], and glaciers [10]. TomoSAR consists of resolving an inversion problem. To date, various spectral analysis methods have been developed for TomoSAR [11] to perform tomography inversion. These methods can be divided into three groups: (1) nonparametric spectral estimation [12,13,14], (2) parametric spectral estimation [15,16,17], and (3) compressive sensing (CS) [18,19,20,21]. Each of the three groups of TomoSAR methods has its respective advantages and drawbacks. For instance, the nonparametric spectral estimators (e.g., beamforming [12,13] and Capon [13,14]) are robust to focusing artifacts but obtain a low height resolution. The parametric spectral estimators, such as the multiple signal classification (MUSIC) estimator [15], the maximum likelihood (ML) estimators [16], and the weighted subspace fitting (WSF) estimators [16,17], can obtain a better height resolution than the nonparametric spectral estimators.

On the other hand, in [22], the single-pass multi-baseline TomoSAR system has 3D resolution, including height-resolving ability in a single-pass platform because there are multiple channels in the cross-track direction. However, the Rayleigh resolution in the height direction is very limited due to the limited baseline length in a single-pass platform. To obtain larger baseline length for better height resolution, the concept of Multistatic SAR [23] was introduced to the TomoSAR research. Moreover, multistatic SAR systems have great application potential in high-resolution wide-swath imaging, moving target detection, interferometric altimetry, 3D/4D imaging, anti-interference, multi-level imaging, etc. Therefore, the concept of a spaceborne multistatic TomoSAR (SMS-TomoSAR) system was proposed. SMS-TomoSAR has multi-baseline, multi-temporal detection capabilities in spatial and temporal dimensions, which can further realize spatial resolution in the height direction, and small surface deformation measurement of the 3D ground scene (i.e., 3D/4D imaging). To date, very limited research has been reported on this aspect of SMS-TomoSAR. Further, a series of key technical issues, such as baseline optimization design, phase error compensation, and imaging processing, directly affect the performance of high-precision resolution imaging in the height direction of SMS-TomoSAR systems.

In this paper, the multi-base multi-pass baselines of SMS-TomoSAR systems are considered equivalent to a group of multi-pass baselines of monostatic SAR. Secondly, the equivalent baselines are established as a symmetric-geometric model to characterize the non-uniform characteristics of baseline distribution. The model is centered on the main image, and baselines on both sides satisfy symmetric-geometric distribution. It is concluded that an approximately uniform baseline distribution has better SMS-TomoSAR imaging performance in the height direction. Further, a baseline design method under uniform-perturbation sampling with normal distribution error is proposed. The imaging performance of different levels of perturbation is compared, and the maximum baseline perturbation allowed by the system is given. Finally, our experimental simulation results verify the effectiveness of the proposed baseline optimization method.

## 2. Baseline Equivalence Analysis of SMS-TomoSAR

**Figure 1:**
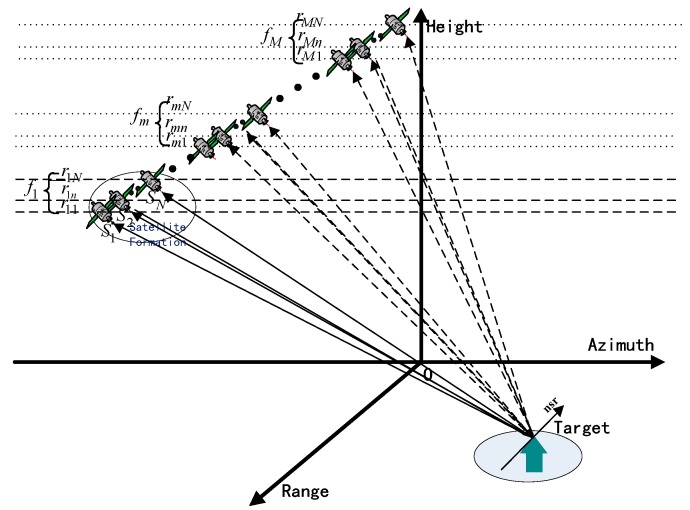
Spatial geometry model of a spaceborne multistatic synthetic aperture radar tomography (SMS-TomoSAR) system (normal-slant-range (nsr) direction).

SMS-TomoSAR systems employ several small satellites $S_{1},S_{2},\ldots ,S_{N}$ in a formation to observe and measure the target area. In particular, SMS-TomoSAR could be considered to be monostatic SAR when an SMS-TomoSAR system employs only one satellite. Compared with monostatic SAR, SMS-TomoSAR obtains more spatial sampling in a single flight, which can reduce the number of revisits and increase the number of samples. Figure 1 shows the spatial geometry model of an SMS-TomoSAR system. The multi-base multi-pass tracks of an SMS-TomoSAR system is expressed as:

(1) $$f_{m}=\left\{r_{mn}|n=1,\;2,\;\ldots ,\;N\right\}m=1,\;2,\;\ldots ,\;M,$$

where n is the nth satellite in the satellite formation, m is the mth flight of the satellite formation, and $f_{m}$ is the track group of the mth flight of the satellite formation.

TomoSAR uses beam-forming technology to obtain high-precision height resolution capability, but tomographic processing requires a large number of spatial samples in different perspectives. Currently, data acquisition systems mainly include single-antenna multi-pass SAR systems, multi-antenna multi-pass In-SAR systems, and single-pass antenna array SAR systems. Essentially, an SMS-TomoSAR system does not differ from other systems in data acquisition, so the multi-base multi-pass baselines of an SMS-TomoSAR system can be considered equivalent to a group of multi-pass baselines of monostatic SAR. Selecting the image obtained by the track $r_{mn}|m=m_{0},n=n_{0}$ as the main image returns NM−1 baselines from NM tracks.

(2) $$\left\{b_{mn}|m\neq m_{0},\;n\neq n_{0}\right\}$$

The baseline in a single flight of a satellite formation is referred to as a same-track baseline, and these same-track baselines of M flights constitute a repeat-track baseline. Considering that there is no essential difference between the baselines of different institutional systems in terms of data acquisition, the repeat-track baseline of SMS-TomoSAR systems is equivalent to:

(3) $$\left\{b_{mn}|m\neq m_{0},\;n\neq n_{0}\right\}=\left\{b_{p}|p=1,\;\ldots ,\;MN-1\right\}.$$

**Figure 2:**
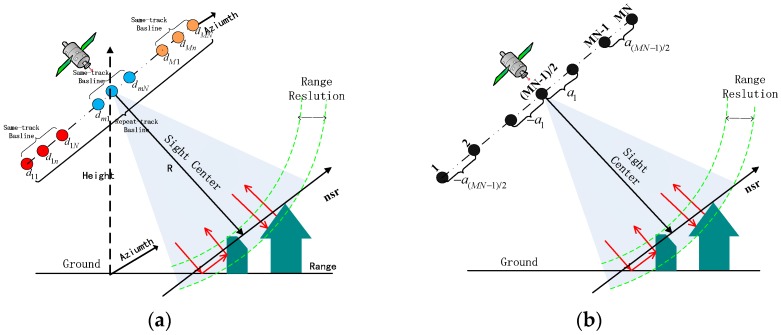
(**a**) Baseline equivalent schematic diagram of an SMS-TomoSAR system; (**b**) Schematic diagram of the symmetric-geometric baseline model.

In addition, the proposed baseline equivalence analysis of an SMS-TomoSAR system is helpful to further explore and obtain the optimal baseline design method. Figure 2a shows the baseline equivalent diagram of an SMS-TomoSAR system.

## 3. Three-dimensional Imaging Analysis of an SMS-TomoSAR System

As shown in Figure 2, an SMS-TomoSAR system observes the same target from different spatial positions, and obtains MN SAR Single Light Complex (SLC) images after azimuth-range two-dimensional compression. The appropriate main image is selected based on factors such as image correlation. After pairing and de-ramping, the complex value of the pth image at the same position [24] is expressed as:

(4) $$g\left(p\right)=\int_{s_{\min}}^{s_{\max}}\gamma (s)\exp\left(j2\pi \xi_{p}s\right)ds\;\;\xi_{p}=2b_{⊥p}/\lambda R,$$

where λ is the radar wavelength; γ(s) is the electromagnetic scattering characteristic function of the target in height domain; R is the distance from the phase center of the radar antenna to the reference point where the height is 0 at the time of capturing the main image; $\left[s_{\min},s_{\max}\right]$ is the height span of the target; $\xi_{p}$ is the spatial frequency corresponding to the height s, and its relationship between the spatial angular frequency is $\xi =\omega_{s}/2\pi$, *b*_⊥*p*_ for the vertical baseline. The specific derivation process is given in [24,25].

Overall, it can be seen from the above derivation process that the complex value of the same-name resolution unit g(p), p=1, 2, …, MN−1 is the discrete sampling in $\xi_{P}$ of the spectrum of the electromagnetic scattering characteristic function γ(s) of the target in the height direction after de-ramping. Therefore, imaging along the height direction is essentially a problem of reconstructing the original signal using spectral discrete sampling, while the vertical baseline $b_{⊥p}$ directly affects system measurement results. Thus, this paper explores the baseline design issues by directly analyzing the vertical baseline, and all baselines discussed below refer to the vertical baseline.

## 4. Spatial Baseline Optimization Method for SMS-TomoSAR systems

### 4.1. Baseline Model Construction

**Figure 3:**
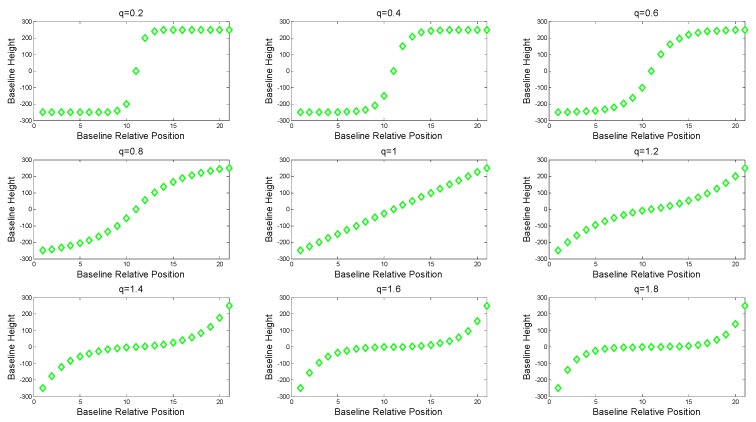
The baseline distribution with changing q (take *MN* = 21 as an example).

Under the given experimental scenario parameters, especially the number of baseline and baseline spans, the effect of the relative intensity of the vertical baseline on the imaging performance of SMS-TomoSAR systems is explored. The vertical baseline $\left\{b_{⊥p}|p=1,\;\ldots ,\;MN-1\right\}$ was planned according to Figure 3. The image centered by the relative position (considered for image correlation and other factors) was selected as the main image, and the baselines on both sides of the main image were assumed to be symmetrically distributed. Taking the (MN−1)/2, …, MN−1, MN images as an example (herein referred to as the Positive Baseline Image Group), the baseline interval of the image group satisfied the equivalent relationship:

(5) $$a_{n}=a_{1}q^{n-1}\;n=1,\;\ldots ,\;(MN-1)/2,$$

(6) $$\frac{B_{span}}{2}=\frac{a_{1}[1-q^{(MN-1)/2}]}{1-q},$$

where q is the baseline interval geometric coefficient, and $B_{span}$ is the baseline span. Since only the positive baseline image group was analyzed here, the positive baseline span was $B_{span}/2$. It can be easily obtained from the above baseline analysis that when q>1, $a_{1}<a_{2}<\cdots <a_{(MN-1)/2}$, that is, the farther from the main image, the more dispersed the baseline distribution. When q<1, $a_{1}>a_{2}>\cdots >a_{(MN-1)/2}$, that is, the farther from the main image, the denser the baseline distribution. Especially, when q=1, $a_{1}=a_{2}=\cdots =a_{(MN-1)/2}$, that is, the baseline of the image satisfies the uniform distribution. Similarly, the above analysis was satisfied for the 1, 2, …, (MN−1)/2 image (i.e., the Negative Baseline Image Group), and will not be discussed again. Figure 3 shows the baseline distribution with varying q.

By adjusting q to change the baseline distribution within the fixed baseline span, image data with different non-uniform characteristics was simulated, and then the imaging and result analysis were performed to explore the influence of different non-uniform characteristics of the baseline on the imaging performance of an SMS-TomoSAR system. Finally, the experimental simulation results showed that an approximate uniform baseline distribution had better SMS-TomoSAR height-dimensional imaging performance.

### 4.2. Maximum Perturbation Estimation Method

To further explore the effect of baseline perturbation on the imaging performance, the experiment defined the ratio of the vertical baseline deviation of the actual track and the predetermined track to double baseline interval under uniform sampling as the baseline perturbation e, and assumed that e was subject to Gaussian distribution, and satisfied

(7) $$e=\frac{\Delta b_{⊥}}{2B_{span}/(MN-1)}∼N\left(0,{\left(e_{\max}/2\right)}^{2}\right),$$

where $\Delta b_{⊥}$ is the vertical baseline deviation and $e_{\max}$ is the maximum baseline perturbation, in which the probability of the vertical baseline perturbation falling in $\left(-e_{\max},\;e_{\max}\right)$ is 99.45%. The actual simulated baseline can be expressed as:

(8) $$\left\{b_{⊥1}+e_{1},\;\ldots ,\;b_{⊥p}+e_{p},\;\ldots ,\;b_{⊥MN-1}+e_{MN-1}\right\},\;p=1,\;\ldots ,\;MN-1,$$

where $\left\{e_{1},\;\ldots ,\;e_{p},\;\ldots ,\;e_{MN-1}\right\},\;p=1,\;\ldots ,\;MN-1$ is a random sample of the baseline perturbation e.

The experiment simulated *T* groups of actual simulated baselines for every maximum vertical baseline perturbation, performed height-dimensional imaging performance analysis, and then used the root mean square error (RMSE) to reflect the impact of the maximum vertical baseline deviation on Peak side lobe ratio (PSLR) and integrated side lobe ratio (ISLR). The RMSE expression on PSLR and ISLR for maximum baseline perturbation e is given as follows:

(9) $$\left\{ \begin{array}{l} RMSE_{PSLR\_e}=\sqrt{\frac{\sum_{i}^{T}{\left(PSLR_{i}-\bar{PSLR}\right)}^{2}}{T}}\\ RMSE_{ISLR\_e}=\sqrt{\frac{\sum_{i}^{T}{\left(ISLR_{i}-\bar{ISLR}\right)}^{2}}{T}} \end{array}\right.$$

where $RMSE_{PSLR\_e}$ and $RMSE_{ISLR\_e}$ are the RMSE values of PSLR and ISLR for e; $PSLR_{i}$ and $ISLR_{i}$ are the PSLR and ISLR values of the ith group of experiments; and $\bar{PSLR}$ and $\bar{ISLR}$ are the mean values of *T* groups of experiments.

The experimental simulation analysis and summary showed that as the baseline perturbation increased, the RMSE value of PSLR and ISLR exhibited a growing trend, that is, the system became more unstable to image in the height direction.

We then define $\sigma_{e}$ as the performance control factor (PCF),

(10) $$\left\{ \begin{array}{l} \sigma_{e\_PSLR}=\frac{RMSE_{PSLR\_e_{\max}}-RMSE_{PSLR\_\min}}{RMSE_{PSLR\_\min}}\\ \sigma_{e\_ISLR}=\frac{RMSE_{ISLR\_e_{\max}}-RMSE_{ISLR\_\min}}{RMSE_{ISLR\_\min}} \end{array}\right.$$

where $\sigma_{e\_PSLR}$ and $\sigma_{e\_ISLR}$ are the PSLR and ISLR performance control factors; $RMSE_{PSLR\_\min}$ and $RMSE_{PSLR\_e_{\max}}$ respectively represent the optimal value and the performance control threshold of PSLR; and similarly, $RMSE_{ISLR\_\min}$ and $RMSE_{ISLR\_e_{\max}}$ respectively represent the optimal value and performance control threshold of ISLR.

We then use

(11) $$e_{\max}=\min(PSLR\_e_{\max},ISLR\_e_{\max})$$

to obtain the maximum allowable baseline perturbation. The experimental simulation showed that the SMS-TomoSAR system had better height-dimensional imaging performance when the baseline met the maximum baseline perturbation.

## 5. Experimental Verification

In this experiment, the proposed baseline analysis model was used to simulate different baseline-intensity images using Matlab software. The experiment was based on the simulation parameters for SMS-TomoSAR systems given in Table 1. The length of the synthetic antenna was 500 m. From Table 1, we know that the experiment simulated 21 baselines. In Figure 1, we define the same-track vertical baseline length $L_{same}$ as the length of vertical baseline interval of an adjacent track in the satellite formation, and the repeat-track vertical baseline length $L_{repeat}$ as the vertical baseline interval of an adjacent track of the same satellite. Compared with an SMS-TomoSAR system, monostatic SAR does not demonstrate same-track vertical baseline length, which directly limits the baseline span of monostatic SAR. In addition, as previously stated, we can consider SMS-TomoSAR as monostatic SAR when an SMS-TomoSAR system employs only one satellite. However, when we specify the number of flights as M=7, the monostatic SAR has only six baselines, which is insufficient to obtain high-precision height resolution images. In practice, we need to obtain enough data to complete an urgent mission within a fixed time, so monostatic SAR is not adequate. Overall, we sought to identify a baseline optimization method for SMS-TomoSAR instead of monostatic SAR, for optimal height-dimensional resolution from one flight of a multi-satellite formation. 

In this paper, we consider the applicability of the optimal baseline design, discrete Fourier transform (DFT), non-uniform discrete Fourier transform (NDFT) and compressive sensing (CS, here in the BP algorithm) imaging algorithms. Further, we compared and analyzed the imaging results in the height direction of those three imaging algorithms for SMS-TomoSAR.

### 5.1. Analysis of Imaging Intensity Results

The experiment set the step size Δq=0.05, and then simulated the satellite image data in q=0.05:2.0, thereby focusing the image through three imaging algorithms: DFT, NDFT, and CS. Given the main image height and baseline span, the Rayleigh resolution of the three imaging algorithms satisfies

(12) rous_DFT=rous_NDFT=rous_CS=15.9m.

Table 2 shows the actual imaging height and phase of three imaging algorithms under nine different q values. The experiment set the target height $S_{0}=8$ and the target phase $\varphi_{0}=45^{\circ }$. It can be seen from Table 2 that all three imaging algorithms could accurately obtain the target height and phase information.

**Figure 4:**
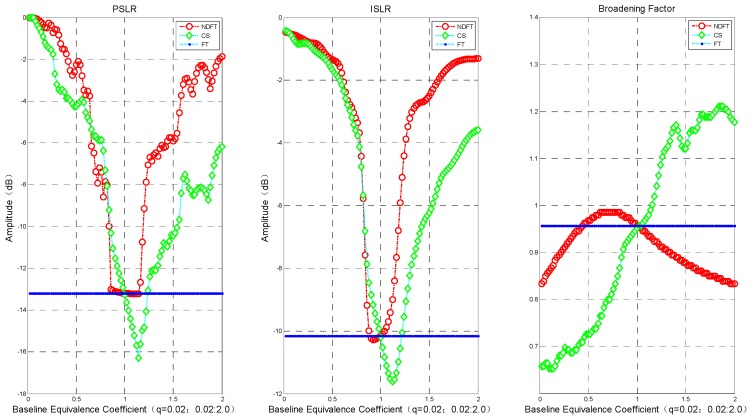
Imaging performance in the height direction: PSLR, ISLR, broadening coefficient. The blue dotted line in the figure only represents the imaging result when the baseline is uniformly distributed.

Figure 4 shows the PSLR, ISLR, and broadening coefficient (actual imaging resolution/Rayleigh resolution) of DFT, NDFT, and CS imaging algorithms under different intensity data. It can be seen from the above results that both the NDFT imaging algorithm and the CS imaging algorithm could achieve and exceed the imaging performance along the height direction of the DFT imaging algorithm when q=1. For the NDFT imaging algorithm, the values of PSLR and ISLR at q=0.86∼1.16 were close to the imaging results of the DFT imaging algorithm, where the actual imaging resolution became gradually worse as q increased. The PSLR and ISLR of the CS imaging algorithm at q=1∼1.22 also achieved and exceeded the imaging results of the DFT imaging algorithm, but with increasing q, the actual imaging resolution gradually became worse.

**Figure 5:**
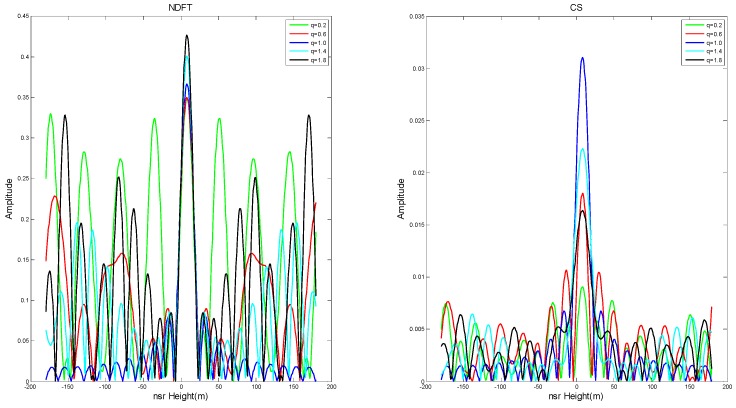
The imaging results of NDFT and CS at *q* = 0.2, 0.6, 1.0, 1.4, and 1.8.

And as |q−1| increased (i.e., the baseline position gradually moved away from or close to the track of the main image), PSLR and ISLR generally increased gradually. In order to ensure a weak target is not obscured by an adjacent strong target, the system requires that the PSLR must be less than 13 dB. It can be seen from Figure 4 and Figure 5 that the imaging algorithm had better imaging performance when the baseline distribution had |q−1| close to 0 (i.e., the baseline tended to be uniformly distributed).

**Figure 6:**
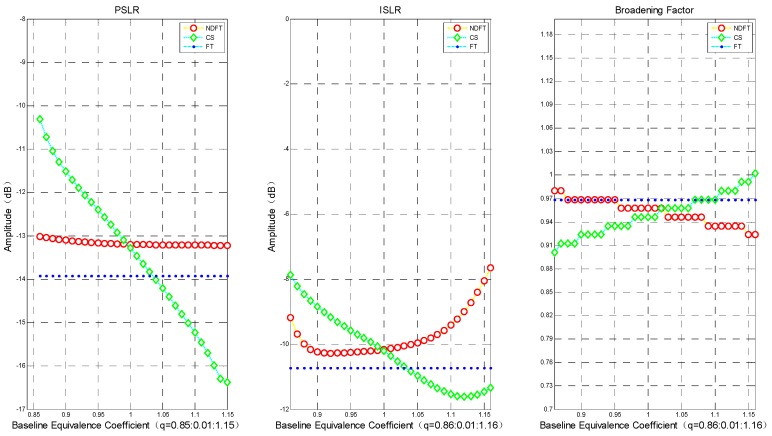
The imaging performance along the deviation direction with q=0.86∼1.16: PSLR, ISLR, broadening coefficient.

In order to further verify the above conclusion, the experiment evaluated the imaging results where PSLR < −10 dB, i.e., q=0.86∼1.16, and took the step size Δq=0.01. The experimental results are shown in Figure 6.

The imaging performance in the height direction of the NDFT and CS imaging algorithms changed gradually. The PSLR of the NDFT imaging algorithm increased at q<0.86, and the imaging performance along the height direction declined sharply when the PSLR of the CS imaging algorithm was greater than −11 dB at q<0.86. Notably, the actual imaging accuracy decreased significantly at q>1.16. At q=0.86∼1.16, the baseline distribution tended to be uniformly distributed, and the broadening coefficient of those three imaging algorithms only varied within ±0.1, as shown in Figure 6. Therefore, summarizing the above analysis results leads to the following conclusion: the system has stable and good imaging performance along the height direction when the baseline satisfies the weak-deviation uniform distribution. We could then use the above conclusion to design the baseline.

### 5.2. Baseline Perturbation Analysis

**Figure 7:**
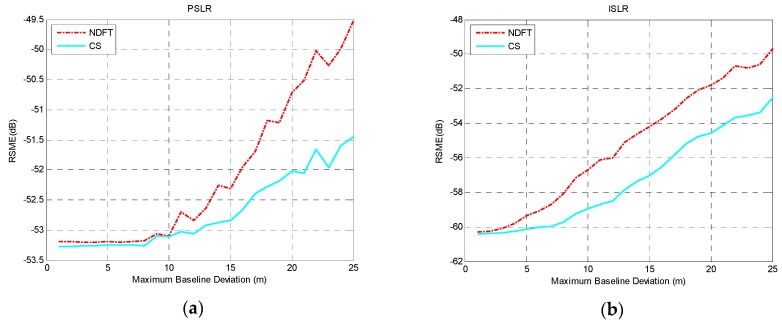
The imaging performance with $B_{span}=500\;m$: (**a**) PSLR; (**b**) ISLR.

**Figure 8:**
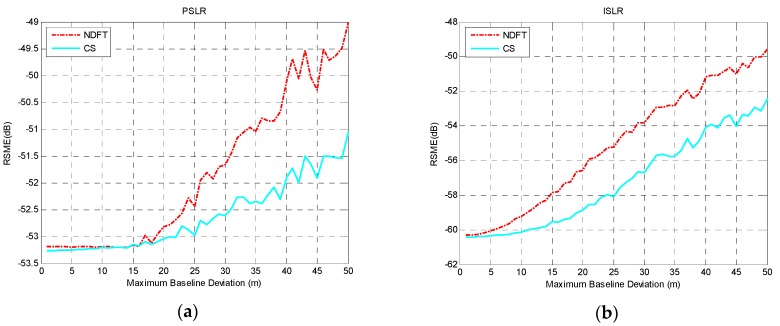
The imaging performance with $B_{span}=1000\;m$: (**a**) PSLR; (**b**) ISLR.

**Figure 9:**
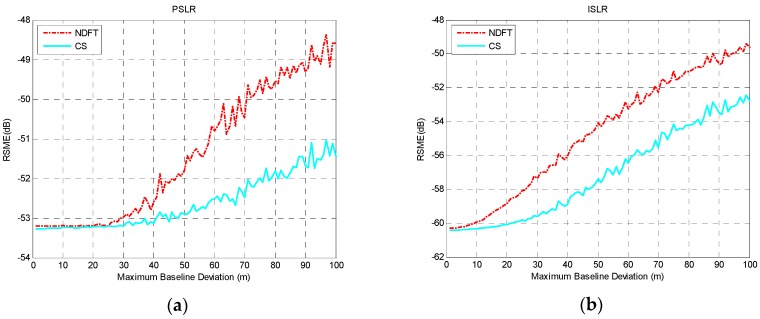
The imaging performance with $B_{span}=2000\;m$: (**a**) PSLR; (**b**) ISLR.

According to the experimental analysis above, when the baseline satisfied uniform sampling within a certain baseline perturbation, the system could achieve optimal imaging performance along the height direction with the aforementioned baseline model. Considering the actual situation of the baseline deviation, $0\leq \Delta b_{⊥}\leq B_{span}/(MN-1)$ was selected and $\Delta b_{⊥}=1\;m$ is the iteration step size, then T=100 and $B_{span}=500,\;1000,\;2000\;m$ were set. The actual baseline was simulated to explore the effect of the maximum baseline deviation on the RMSE value of PSLR and ISLR. The results are shown in Figure 7, Figure 8 and Figure 9.

It can be seen from the experimental results that the height-dimensional imaging performance of the system was basically stable within a certain baseline perturbation, and as baseline deviation increased, the height-dimensional imaging performance was reduced, which can be seen from the mean and variance of PSLR and ISLR.

In order to ensure adequate imaging performance within the baseline deviation, the experiment selected $\sigma_{e\_PSLR}=0.004$ and $\sigma_{e\_PSLR}=0.04$ to obtain the result of the maximum baseline deviation with $B_{span}=500\;m,\;1000\;m,\;2000\;m$, as shown in Table 3.

It can be seen from the above results that as the baseline span increased, the maximum baseline perturbation of the imaging performance increased linearly.

**Figure 10:**
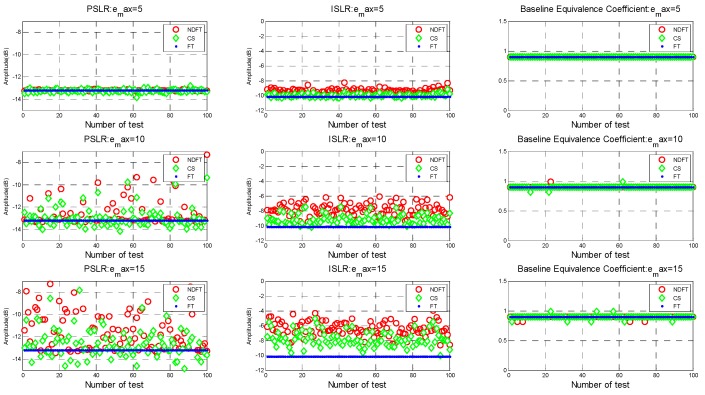
Imaging performance results when $e_{\max}=10\%,\;20\%,\;40\%$.

The results of generating 100 sets of baseline with $B_{span}=500\;m$ and $e_{\max}=10\%,\;20\%,\;40\%$, and subsequent height-dimensional imaging performances are shown in Figure 10.

It can be seen from the above experimental results that the imaging performance was more unstable as the maximum baseline perturbation $e_{\max}$ increased (i.e., the farther the baseline deviated from the predetermined uniform baseline).

### 5.3. Baseline Optimization Method Verification

**Figure 11:**
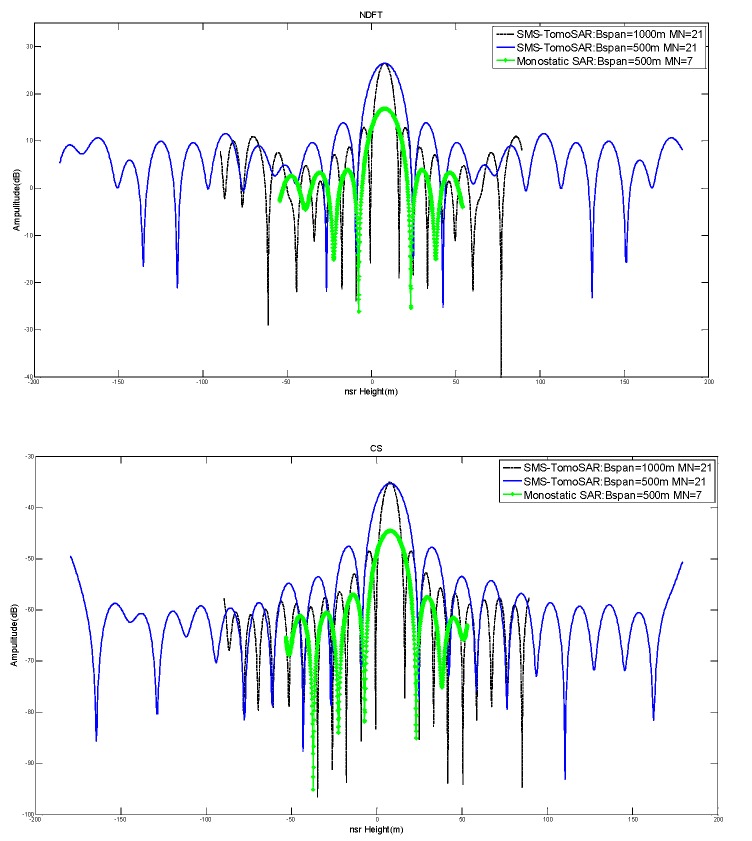
The imaging results of SMS-TomoSAR and monostatic SAR.

As mentioned above, we can consider SMS-TomoSAR to be monostatic SAR, when N=1. However, monostatic SAR does not exhibit the same-track vertical baseline length, which directly limits the baseline span of monostatic SAR. However, when given the number of flights M=7, monostatic SAR has only six baselines, which is insufficient to image for high-precision height resolution. In Figure 11 and Table 4, based on the parameters in Table 1 and q=1.0, e=20%, the performance results of SMS-TomoSAR and monostatic SAR are given. The experiment showed only the results of monostatic SAR under $S_{span}=500\;m$ due to the limited baseline span for the same-track vertical baseline length.

From the results above, compared with SMS-TomoSAR, it is obvious that monostatic SAR had smaller height span and fewer baselines. This directly resulted in lower unambiguity height and worse PSLR and ISLR. For an urgent mission within a fixed time, monostatic SAR is not suitable. The proposed method could be used if monostatic SAR obtains the same number of baselines as SMS-TomoSAR by more flights. However, SMS-TomoSAR and monostatic SAR have different influences on some issues, such as spatial transmission error estimation and compensation, as well as deformation measurement. Consequently, in this paper, we focus mainly on the effect of the proposed method for SMS-TomoSAR.

**Figure 12:**
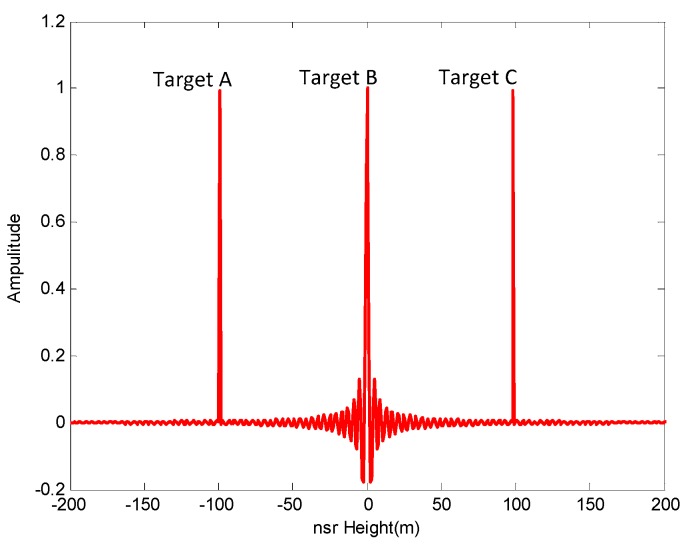
Simulated target.

To further verify the validity, we used simulated data to provide tomographic images of scatterers located in the same cross-slant-range line, and obtained the image by inverting the model using the described techniques. Firstly, three isolated and coherent targets A, B, and C were simulated, and then an A and C simulated coherent targets located on the bottom and on the top, respectively, gave a distributed target B with a certain extension, but separated from the coherent target located on the bottom. The simulated target information is seen in Table 5 and Figure 12.

**Figure 13:**
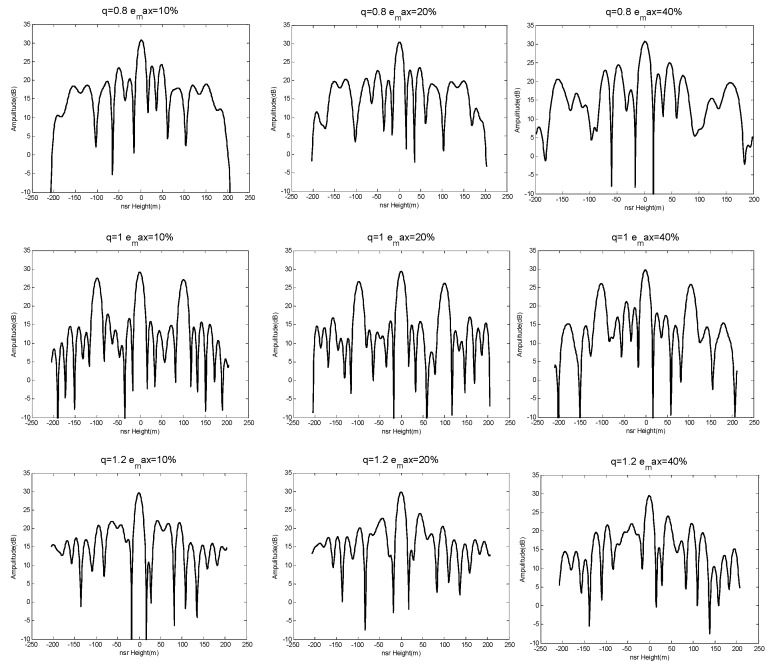
Results of the NDFT algorithm.

**Figure 14:**
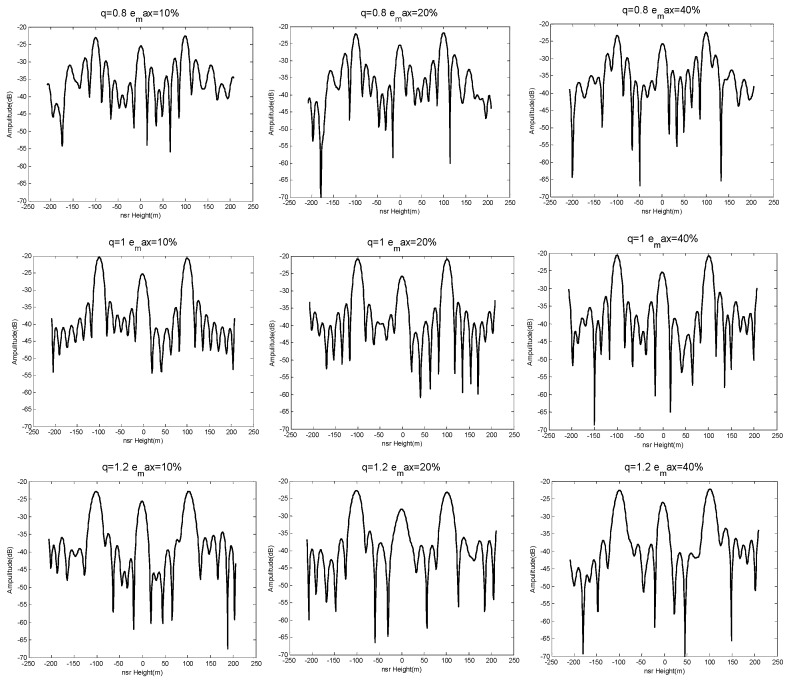
Results of the CS algorithm.

According to the conclusion above, we selected q=0.8, 1.0, 1.2 and $e_{\max}=10\%,\;20\%,\;40\%$ based on Table 1. The simulation results are seen in Figure 13 and Figure 14.

We can conclude from the results shown in Figure 13 and Figure 14 that the NDFT and CS algorithms were better able to distinguish those three simulated targets when $q=1.0,\;e_{\max}=10\%$. This verifies the system had stable and good imaging performance in the height direction when the baseline satisfied a weak-deviation uniform distribution. Furthermore, the system had superior height-orientation performance when the baseline of an SMS-TomoSAR system met the allowable maximum baseline perturbation. In other words, the allowable maximum baseline perturbation can provide a reference satellite baseline control deviation for the design or spatial position control of SMS-TomoSAR systems. For example, when the experiment set $S_{span}=500\;m$, MN=7 with the NDFT imaging algorithm, a maximum baseline perturbation provided optimal height-orientation performance for SMS-TomoSAR systems.

## 6. Conclusions

In this paper, we established a symmetric-geometric baseline analysis model to explore the effect of baseline intensity on the height-dimensional imaging performance within a fixed baseline span, and proposed a maximum baseline deviation estimation method to explore the maximum vertical baseline deviation that ensures adequate height-dimensional imaging performance. From the experimental results above, it can be concluded that the system had superior height-orientation performance when the baseline of an SMS-TomoSAR system met the allowable maximum baseline perturbation, which has practical significance for the actual system baseline design. In other words, the allowable maximum baseline perturbation can provide a reference satellite baseline control deviation for the design or spatial position control of an SMS-TomoSAR system. Finally, we will continue to investigate other issues of SMS-TomoSAR systems, in order to contribute further to the existing body of research in the future.

## Figures and Tables

**Table 1:** Simulation parameters.

Parameter	Value	Parameter	Value
Main image height (m)	H=600,000	Target height (m)	$S_{0}=8$
Number of formation satellites	N=3	Target complex scattering coefficient	γ(s)=exp(jπ/4)
Number of flights	M=7	Target height span (m)	$\left[s_{\min},s_{\max}\right]=[-180,180]$
Repeat-track vertical baseline length (m)	$50\leq L_{repeat}\leq 100$	Same-track vertical baseline length (m)	$100\leq L_{same}\leq 200$
Baseline span (m)	$B_{span}=500$	Unambiguity height (m)	$S_{unambiguity}=360$

**Table 2:** Actual imaging height error and phase. CS: compressive sensing; DFT: discrete Fourier transform; NDFT: non-uniform discrete Fourier transform.

	DFT	NDFT	CS
q=0.2		0.42 m/45.0°	0.42 m/45.0°
q=0.4		0.42 m/45.0°	0.33 m/45.0°
q=0.6		0.42 m/45.0°	0.29 m/45.0°
q=0.8		0.42 m/45.0°	0.42 m/45.0°
q=1.0	0.42 m/45.0°	0.42 m/45.0°	0.42 m/45.0°
q=1.2		0.42 m/45.0°	0.42 m/45.0°
q=1.4		0.42 m/45.0°	0.42 m/45.0°
q=1.6		0.42 m/45.0°	0.11 m/45.0°
q=1.8		0.42 m/45.0°	0.42 m/45.0°

**Table 3:** The relationship of baseline span and critical deviation about the performance stability.

Parameter	$B_{span}=500\;m$	$B_{span}=1000\;m$	$B_{span}=2000\;m$
$NDFT:PSLR\_e_{\max}$	18.0%	17.0%	15.0%
$NDFT:ISLR\_e_{\max}$	14.0%	14.0%	12.5%
$NDFT:e_{\max}$	14.0%	14.0%	12.5%
$CS:PSLR\_e_{\max}$	20.0%	20.0%	21.5%
$CS:ISLR\_e_{\max}$	22.0%	23.0%	22.5%
$CS:e_{\max}$	20.0%	20.0%	21.5%

**Table 4:** The imaging performance results of SMS-TomoSAR and monostatic SAR for the NDFT and CS algorithms.

Parameter (NDFT/CS)	Monostatic SAR $\left(N=1\;B_{span}=500\;m\right)$	SMS-TomoSAR $\left(N=3\;B_{span}=500\;m\right)$	SMS-TomoSAR $\left(N=3\;B_{span}=1000\;m\right)$
Target height span (m)	[−54.3,54.3] /[−53.2,53.2]	[−184.7,184.7] /[−179.4,179.4]	[−89.5,89.5] /[−89.4,89.4]
Rayleigh resolution (m)	16.02 m/15.71 m	16.36 m/15.90 m	7.93 m/7.92 m
Imaging height error and phase	(0.13 m/45.0°) /(0.08 m/45.0°)	(0.49 m/45.0°) /(0.24 m/45.0°)	(0.23 m/45.0°) /(0.21 m/45.0°)
Unambiguity height (m)	108.5 m/106.4 m	369.4 m/358.8 m	179.0 m/178.7 m
PSLR (dB)	−0.000317/−0.000282	−12.62468/−12.31986	−13.60256/−13.40242
ISLR (dB)	−0.031460/−0.032658	−6.506870/−8.981114	−8.034666/−10.06845

**Table 5:** Target information.

Target	Complex Scattering Coefficient	Target nsr Height (m)
Target A	$\gamma_{A}(s)=\exp(j\pi /4)$	$S_{0}=-100$
Target B	$\gamma_{B}(s)=\exp(j\pi /4)*\sin c(s)$	$S_{0}=0$
Target C complex scattering coefficient	γ(s)=exp(jπ/4)	$S_{0}=100$
